# Drug resistance against 5-fluorouracil and cisplatin in the treatment of head and neck squamous cell carcinoma: A systematic review

**DOI:** 10.34172/joddd.2021.036

**Published:** 2021-08-25

**Authors:** Fatemeh Atashi, Nafiseh Vahed, Parya Emamverdizadeh, Shirin Fattahi, Ladan Paya

**Affiliations:** ^1^Private Practice, Tabriz, Iran; ^2^Research Center for Evidence-based Medicine, Tabriz University of Medical Sciences, Tabriz, Iran; ^3^Department of Oral and Maxillofacial Pathology, Faculty of Dentistry, Tabriz University of Medical Sciences, Tabriz, Iran

**Keywords:** 5-fluorouracil resistance, Chemoradiation resistance, Cisplatin resistance, Drug resistance, Head and neck squamous cell carcinoma

## Abstract

Head and neck cancers are highly prevalent worldwide. Most of these lesions are diagnosed in the advanced stages of the disease. Thus, they do not often have a good long-term prognosis. Like other cancer types, head and neck cancers are managed by surgery, radiotherapy, and chemotherapy. Despite significant advances in the treatment of oral squamous cell carcinoma (OSCC), physicians encounter several challenges in the course of treatment. Various mechanisms mediate the clinical responses of a certain cancer to medications. Thus, efficient treatment planning requires adequate knowledge about the genes involved in drug resistance and the evaluation of the frequency percentage of resistance. Several studies have evaluated the causes and frequency percentages of 5-fluorouracil (5-FU) and cisplatin resistance. In this systematic review, all the relevant articles published until November 30, 2019, were retrieved from the Scopus, Embase, Medline, ISI, Web of Science, and Cochrane databases using certain MeSH and EMTTree keywords. A total of 2164 articles were retrieved of which, 18 were included in the review since they had reported the frequency percentages of drug resistance. Of all, 10 articles had evaluated cisplatin (1317 samples). A meta-analysis of the results revealed a frequency of 33% for cisplatin resistance. Eight studies had evaluated 5-FU (476 samples). A meta-analysis of the results revealed a frequency of 40.2 % for 5-FU resistance. Overcoming cisplatin resistance or 5-FU resistance can significantly enhance recovery in advanced HNSCC. Attempts should be made to eliminate the cause and use multi-drug regimens to increase the success rate of treatment.

## Introduction

Head and neck squamous cell carcinoma (HNSCC) is the sixth most common cancer worldwide, which can involve the oral cavity, lips, nose, sinuses, larynx, and pharynx.^1^ Squamous cell carcinoma (SCC) accounts for 2% and 4% of all cancer types in females and males, respectively.^2^ Similar to other cancer types, SCC is managed by surgery, radiotherapy, and chemotherapy. Surgical resection is the standard treatment modality for SCC. Chemotherapy is often performed for patients with advanced or recurrent oral SCC (OSCC).^3^ Despite the advances in the rate of early diagnosis, multi-drug treatments, and surgical interventions, the overall 5-year survival rate of patients with advanced HNSCC is still low (< 25%) and has remained almost unchanged in the past 30 years.^4^ Emergence of drug resistance in the tumor often leads to treatment inefficacy. Statistical data indicate that drug resistance is responsible for over 90% of mortalities and morbidities in cancer patients.^5^

Cisplatin, also known as cis-dichlorodiamine platinum, belongs to the platinum-based antineoplastic family of medications.^6^ Cisplatin has anti-cancer properties and is a cell-cycle nonspecific antineoplastic agent. It can impair the synthesis of the DNA purine base and subsequently cause DNA damage and eventual apoptosis of cancer cells.^7^ Although cisplatin is initially successful in 80%‒90% of patients, cancer cells eventually develop resistance to it. Cisplatin resistance occurs in approximately one-third of women during their course of treatment.^6^

Five-fluorouracil (5-FU) is another anti-cancer medication prescribed to treat several cancer types, such as gastric cancer, colon cancer, lung cancer, breast cancer, and head and neck cancers.^8^ The DNA of cancer cells easily uptakes this medication since it produces uracil. Five-fluorouracil is metabolized to 5-fluorodeoxyuridylate, and suppresses the synthesis of tumoral DNA. Also, 5-FU is converted to 5-fluorouridine triphosphate, which is incorporated in the structure of RNA and inhibits the synthesis of tumoral RNA.^9^

Problems related to the emergence of drug resistance to 5-FU are the main obstacles against cancer treatment. Thus, there is an immediate need for further elucidation of important molecular pathways involved in the development of 5-FU resistance to enhance the efficacy of chemotherapy and improve the treatment prognosis.^10^

Considering the high rate of morbidity and mortality due to OSCC, high cost of treatment, the imposed burden on the society, and lack of comprehensive systematic reviews on this topic, this systematic review aimed to comprehensively assess drug resistance to cisplatin and 5-FU in the treatment of HNSCC.

## Methods

This systematic review was conducted at the Evidence-based Medical Research Center and Faculty of Dentistry, Tabriz University of Medical Sciences in 2020 on 5-FU resistance and cisplatin resistance in the treatment of HNSCC. The Embase, Scopus, Medline, ISI Web of Science, and Cochrane databases were searched for relevant English articles published from January 1970 to November 30, 2019. The reference lists of the retrieved articles were also searched manually. The grey literature and conference papers were also searched. Moreover, researchers with a research background on this topic were contacted to find published and unpublished articles on the topic. The searched keywords included head and neck SCC patients, oral SCC, patients receiving cisplatin, patients receiving 5-FU, SCC medication, drug resistance, cisplatin resistance, 5-FU resistance, oral diseases, cancer treatment medicine, and their combinations.

### Study selection

The inclusion criteria were articles published in English, descriptive studies, clinical trials, cross-sectional studies, cohort studies, and case series, articles published from 1970 to the end of 2019, and conference papers.

The exclusion criteria were non-English articles, low-quality articles, duplicated articles (risk of bias), review articles, letters to editors, and editorials.

### Assessment of studies

The articles retrieved from the search were evaluated in several steps, and their titles, abstracts, and full-texts were screened. Articles that met the eligibility criteria were included in the study. The titles were first evaluated, and accordingly, ambiguous cases and those not meeting the study objectives were excluded. The abstracts of the articles were then read, and those meeting the eligibility criteria were processed for data extraction. Next, the full-texts of the articles, their methodology, and results were studied. Controversies were evaluated, and the grey literature was also assessed and reported.

Using the Joanna Briggs Institute checklist for evaluating the quality of articles, the contents of the manuscripts and their quality were assessed by two independent examiners according to the existing standards. Low-quality articles were excluded.

### Statistical analysis

The frequency percentages of drug resistance were extracted from the included studies. To determine the pooled frequency percentages of drug resistance, a meta-analysis was performed using the random-effect model. To assess the heterogeneity of the studies, the I^2^ and chi-square tests were used. Subgroup analysis based on the type of medication was also performed. A funnel plot was drawn to check the publication bias, and the Egger’s regression test was also applied. The Trim and Fill method was used to control for the publication bias and determine the number of missed articles. *P* < 0.05 was considered statistically significant.

## Results

### Search Results and Characteristics of Studies

**Figure 1:**
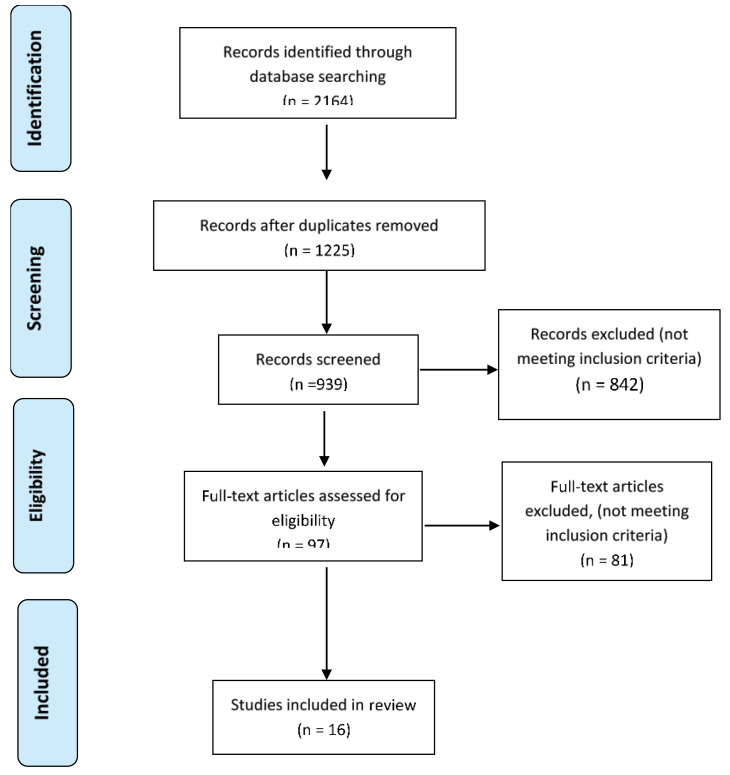
Search strategy and selection process of the systematic review.

The systematic search yielded 2164 articles, of which 939 were excluded since they were duplicates. Also, 383 articles were excluded after evaluating their titles and abstracts. After reviewing the full-texts of the remaining articles, 745 were excluded. Eventually, 16 articles underwent meta-analysis. Studies by Luo et al^11^ and Bauer et al^12^ were considered as four separate studies since they had evaluated both medications. Of selected studies, 8 studies^13-20^ had evaluated cisplatin, six studies^21-26^ had evaluated 5-FU, and two studies^11,12^ had evaluated both medications. The flowchart for the literature search and article selection is shown in Figure 1.

The articles were first arranged based on their publication date, and then the first author’s name, publication year, sample size, the gender of patients, type of medication used, medication dose, and frequency percentage of drug resistance, were collected. Table 1 presents the characteristics of the studies subjected to meta-analysis.

**Table 1:** Characteristics of the studies subjected to meta-analysis

**Author’s name, publication year**	**Sample size**	**Age**	**Gender**		**Dosage (mg/m** ^2^ **)**	**Duration of use (months)**	**Drug type**	**Cancer grade**				**Percentage of drug resistance**
			**Male**	**Female**				**I**	**II**	**III**	**VI**	
Wang et al (2019)	14					7	Cisplatin					37%
Hsu et al (2019)	502		350	152	100	2	Cisplatin					47%
Zhang et al (2018)	510	60	375	135		8	Cisplatin	26	69	81	260	17%
Feng et al (2017)	103	60	30	30			5-FU	39	64	64		68.9%
Yang et al (2016)	43	59	43	0			Cisplatin					35.7%
Nakamura et al (2015)	49	55.5	27	22	120	1	5-FU	4	4	15	15	38.7%
Luo et al (2015)	121	50.4	117	4	75		Cisplatin					37%
Luo et al (2015)	121	50.4	117	4	1000		5-FU				121	37%
Basak et al (2015)	5				100		Cisplatin					25%
Chang et al (2014)	103		83	20			Cisplatin	7	46	50		30%
Kawakara et al (2013)	61	65	37	24	120	60	5-FU		13	20	28	49%
Ota et al (2012)	7					6	Cisplatin					30%
Nagata et al (2011)	54	65	31	23		24	5-FU		4	19	31	27%
Chiu et al (2011)	57	50	55	2	1000	24	5-FU				57	39%
Bauer et al (2005)	2						Cisplatin					40%
Bauer et al (2005)	2						5-FU					40%
Cullen et al (2003)	10						Cisplatin					20%
Akervall et al (2004)	29		23	6	1000		5-FU		1	11	17	40%

### Meta-analysis results

**Figure 2:**
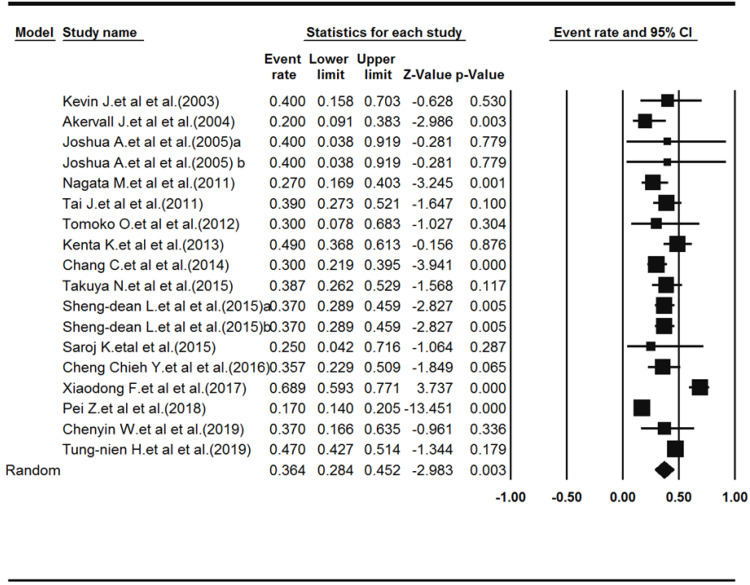
Forest plot diagram of drug resistance prevalence.

Eighteen studies reported drug resistance (a total of 1793 samples). The mean age of patients had been reported in 9 studies, which was 57.25 years. A total of 1288 males and 422 females had been evaluated by the 12 studies that had reported the gender of patients. Heterogeneity among the studies was significant (Q-value = 152.168, df-value = 17, I^2^ = 88.82, *P* < 0.001). According to the results of the meta-analysis, the prevalence of drug resistance was 36.4% (pooled resistance = 0.364, 95% CI: 0.284‒0.452, *P* = 0.003). Figure 2 shows the Forest plot of the results.

**Figure 3:**
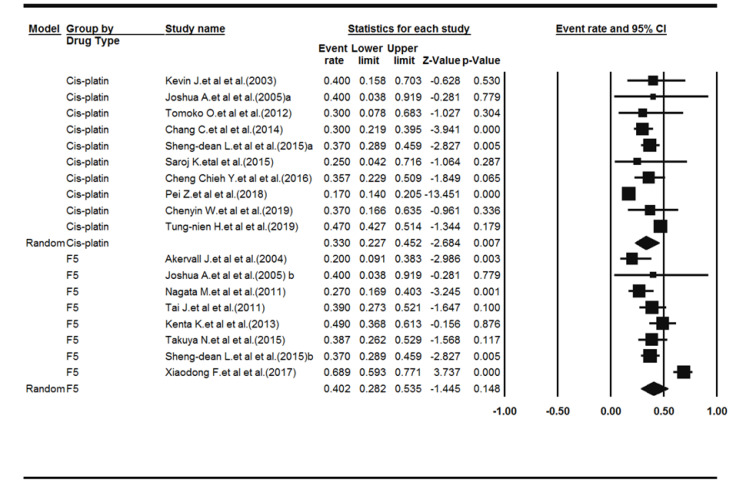
Forest plot diagram of drug resistance prevalence based on the type of medication.

Subgroup analysis was then performed based on the type of medication used. Ten studies had evaluated cisplatin (1317 samples). Heterogeneity among the studies was significant (Q-value = 99.83, df-value = 9, I^2^ = 90.98, *P* < 0.001). The results of the meta-analysis showed a frequency percentage of 33% for cisplatin resistance (pooled resistance = 0.330, 95% CI: 0.227‒0.452, *P* < 0.007). Eight studies had evaluated 5-FU (476 samples). Heterogeneity among the studies was significant (Q-value = 39.98, df-value = 7, I^2^ = 82.49, *P* < 0.001). The results of the meta-analysis showed a frequency percentage of 40.2% for 5-FU resistance (pooled resistance = 0.402, 95% CI: 0.282‒0.536, *P* < 0.148). Figure 3 shows the Forest plot of subgroup analysis.

### Release bias (publication bias)

**Figure 4:**
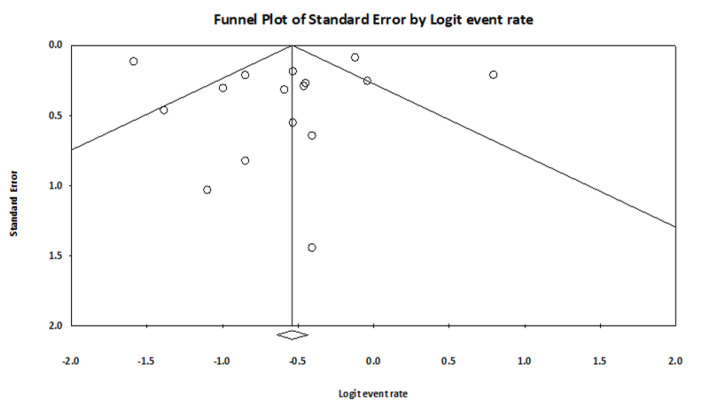
Funnel diagrams for the evaluation of diffusion bias in studies included in the meta-analysis

Funnel diagram and Egger’s regression test were used to investigate the diffusion bias between studies included in the meta-analysis. Figure 4 shows the funnel diagram. According to the funnel diagram, there is no significant asymmetry. Also, according to the regression results, the diffusion between studies included in the meta-analysis was not statistically significant (t-value = 0.097, df = 16, *P* value = 0.91)

## Discussion

At present, chemotherapy, radiotherapy, and surgery are the most common therapeutic approaches for cancer management. Chemotherapy is the first line of treatment for lymphoma, leukemia, and lung cancer. For other solid tumors, chemotherapy can serve as an adjunct for eliminating residual nodes after the surgical procedure to minimize the risk of tumor recurrence or before surgery or radiotherapy.^27^ Nonetheless, many cancer types develop resistance to chemotherapeutic agents. Several mechanisms have been suggested explaining drug resistance. Among the studies that reported the frequency percentage of drug resistance, two studies reported that the reason for the development of drug resistance was the mutation of cancer cells^14,26^ and tried to find strategies to prevent such mutations. Another study found that the expression of osteopontin resulted in poor response to treatment and survival of cancer cells.^11,22^ Since the medications used in the aforementioned two studies were different, it might be concluded that the type of drug resistance cannot be exclusive to one type of medication. Another cause of the emergence of drug resistance is the increased expression of pro-inflammatory cytokines in the path of drug uptake by cancer cells, which affects the drug efficacy.^16,21^ Bauer et al^28^ reported that drug resistance is due to the low level of P53 and high level of Bcl-xL. To overcome the interactions of P53, an MDM2 inhibitor was used as a new treatment strategy, which reactivated the function of P53 in HNSCC cells with the wild-type P53 and impaired the cell cycle, leading to cancer cell apoptosis. Designing and developing inhibitors of small non-peptide molecules to target the interactions of P53 is an interesting novel treatment strategy for the treatment of cisplatin-resistant HNSCC with wild-type P53.

Akhter and Enamur Rashid analyzed the expression of thymidylate synthase and dihydropyrimidine dehydrogenase in the treatment of OSCC with 5-FU in 50 patients and concluded that over-expression of thymidylate synthase played a significant role in 5-FU resistance. Also, the inhibition of tumoral dihydropyrimidine dehydrogenase increased the sensitivity threshold of cancer cells to this medication.^29^ Gao et al^30^ evaluated mRNA expression by the cancer cells in 399 patients with HNSCC and showed that high expression of interleukin 6 was associated with poor prognosis and chemical resistance in many cancer cells, without requiring any mediator.

In the present study, the results of the meta-analysis regarding cisplatin resistance revealed a frequency percentage of 33%. Of the 18 studies evaluated, the one by Yang et al^16^ reported a 35.7% frequency percentage of cisplatin resistance in 43 tissue specimens of patients, which was close to the frequency percentage reported in our study. The results of the meta-analysis reported the frequency percentage of 5-FU resistance to be 40.2%, which was close to the 40% rate reported by Bauer et al.^12^

The mean age of patients was 25.57 years in the nine studies that reported the age of patients in this systematic review. Among all, the mean age reported by Yang et al,^16^ which was 59 years, and the mean age reported by Nakamura et al,^22^ which was 55.5 years, were closer to our results. In our study, the percentage of patients taking cisplatin was higher than patients using 5-FU; nonetheless, the mean frequency percentage of cisplatin resistance was lower than that of 5-FU resistance. Chen et al^31^ compared the treatment results and toxicity of cisplatin alone and cisplatin plus 5-FU for treating HNSCC and concluded that postoperative chemotherapy with cisplatin alone resulted in a higher 3-year survival rate and lower treatment complications and side effects compared with cisplatin plus 5-FU.

This study was the first to perform a meta-analysis on cisplatin and 5-FU resistance. According to the results, most studies on cancer treatment with chemotherapy do not report the percentage of drug resistance and mainly focus on the cause of the emergence of drug resistance. On the other hand, in most studies that reported the percentage of drug resistance, the frequency percentage of drug resistance was not significantly correlated with the severity of disease because the majority of the tested samples were in the end stage of the disease. Also, an unequal number of male and female patients was another limitation of the reviewed studies; thus, the correlation between the frequency percentage of drug resistance and gender could not be analyzed.

## Conclusion

In the present study, the frequency percentages of cisplatin resistance and 5-FU resistance were 33% and 40.2%, respectively. Accordingly, it might be concluded that cisplatin, which is the basis of chemotherapy regimens for HNSCC, is not effective alone in many patients, and cisplatin-based regimens might result in suboptimal disease control. Combination therapy with other medications might be more effective. Overcoming cisplatin or 5-FU resistance can significantly improve the prognosis of treatment of advanced HNSCC. On the other hand, further studies on the causes of drug resistance can pave the way for more successful multi-drug treatments.

## Authors’ Contributions

FA, NV, and PE contributed in the design of the study. SF and LP contributed to the data search. All the authors participated in manuscript preparation and revision.

## Acknowledgments

The authors thank the Research Center for Evidence-based Medicine of Tabriz University of Medical Sciences for financial support.

## Funding

This paper was extracted from a thesis and financially supported by the Research Center for Evidence-based Medicine of the Tabriz University of Medical Sciences.

## Competing Interests

The authors declare no conflict(s) of interest related to the publication of this work.

## Ethics Approval

This systematic review was carried out based on the Preferred Reporting Items for Systematic Reviews and Meta-Analyses (PRISMA) guidelines for reporting systematic reviews. The research protocol was approved by the Ethics Committee of Tabriz University of Medical Sciences (IR.TBZMED.VCR.REC.1398.331).
